# Exploring cortex in a high-throughput manner by building brain observatories

**DOI:** 10.1186/1471-2202-15-S1-A2

**Published:** 2014-07-21

**Authors:** Christof Koch

**Affiliations:** 1 Allen Institute for Brain Science, Seattle, WA 98103, USA

## 

The *Allen Institute for Brain Science* has, over the past ten years, produced a series of brain atlases (http://www.brain-map.org). These are large (3 TB, >1 million slides) public resources, integrating genome-wide gene expression, and neuroanatomical data across the entire brain for developing and adult humans, non-human primates and mice, complemented by high-resolution, cellular-based anatomical connectivity data in several thousand mice. It is the single largest integrated neuroscience database world-wide. Anybody can freely access this data without any restrictions.

We are embarked on an ambitious 10-year initiative to understand the structure and function of the neocortex and associated satellite structures in humans and mice. We are setting up high through-put pipelines to exhaustively characterize the morphology, electrophysiology and transcriptome of cell types as well as their synaptic interconnections in the human neocortex (via a combination of fetal, neurosurgical and post-mortem tissues & human stem cells differentiated into forebrain neurons) and in the laboratory mouse. We are building brain observatories to image the activities of neurons throughout the cortico-thalamic system in behaving mice, to record their electrical activities, and to analyze their connectivity at the ultra-structural level. We are constructing biophysically detailed as well as simplified computer simulations of these networks and of their information processing capabilities. In keeping with the Allen Institute for Brain Science’s core value of open science, all data, knowledge and tools from this initiative will be shared with the broader scientific community.

